# Novel Sweat-Based Wearable Device for Advanced Monitoring of Athletic Physiological Biometrics

**DOI:** 10.3390/s23239473

**Published:** 2023-11-28

**Authors:** Javier Aguilar-Torán, Genis Rabost-Garcia, Samantha Toinga-Villafuerte, Albert Álvarez-Carulla, Valeria Colmena-Rubil, Andrea Fajardo-Garcia, Andrea Cardona-Bonet, Jasmina Casals-Terré, Xavier Muñoz-Pascual, Pere Miribel-Català, Jaime Punter-Villagrasa

**Affiliations:** 1 Onalabs Inno-Hub SL, 08290 Cerdanyola del Vallès, Spain; 2 Department of Electronics and Biomedical Engineering, Barcelona University, 08028 Barcelona, Spain; 3 Department of Mechanical Engineering, Polytechnic University of Catalonia, 08222 Terrassa, Spain; 4 Department of Chemistry, Autonomous University of Barcelona, 08193 Cerdanyola del Vallès, Spain

**Keywords:** sweat monitoring, perspiration level, dehydration, lactate, heart rate, wearable device, sports medicine

## Abstract

Blood testing has traditionally been the gold standard for the physiological analysis and monitoring of professional athletes. In recent years, blood testing has moved out of the laboratory thanks to portable handheld devices, such as lactate meters. However, despite its usefulness and widespread use, blood testing has several drawbacks and limitations, such as the need for the athlete to stop exercising for blood extraction and the inability to have data continuously collected. In this scenario, sweat has become an alternative to blood testing because of its rich content of electrolytes and metabolites, as well as small quantities of sugars, proteins, and ions. Nevertheless, there are few devices capable of analyzing this biofluid and providing useful information to users. In this paper, an electronic system designed for the autonomous analysis of sweat electrolytes and metabolites along with heart rate dynamics is presented. This system is part of a novel wearable device tailored for athletes that offers to the user a real-time assessment of their physiological status and performance.

## 1. Introduction

Blood tests are regularly used by clinicians to assess the state of health of patients. This trial is the gold standard for biochemical analyses, but it has several drawbacks that limit its use. Blood tests are invasive, require trained personnel, and only provide the state of the patient at the moment of the extraction. An alternative analysis that has gained a lot of interest during the past few years is based on the use of sweat as the sample to be analyzed [1,2].

Sweat is a biofluid composed mainly of water, but it contains various electrolytes, ions, amino acids, proteins, and other small molecules. Due to its rich content, this fluid has become a great candidate for analysis. Sweat analysis is non-invasive, does not require a professional to collect the sample, and can be carried out continuously, since this biofluid is always present on the skin due to the body’s thermoregulation [3]. 

Despite these advantages, nowadays, sweat is barely used, and only some trials, such as the cystic fibrosis test, utilize this biofluid [4]. The lack of tests that use sweat as a sample is because a standardized method to extract sweat from skin does not exist. This fact, along with the low volume of the sample available (range of nanoliters) and liquid evaporation, makes sweat a biofluid that is difficult to measure. These issues are solved by attaching sensors to the skin, but this methodology produces a major issue: contamination of the sample due to the accumulation of sweat on the sensor. To avoid this unwanted situation, a microfluidic system can be used to renew the sample and expel the generated waste [5].

Several research groups have developed systems capable of collecting sweat from the skin, processing it, and analyzing its content using different sensors. Liang et al. developed a paper-based microfluidic device for the real-time monitoring of sweat potassium [6]. Martín et al. presented a microfluidic detection platform for the monitoring of glucose and lactate levels [7]. Kim et al. developed a microfluidic patch that allowed for rapid colorimetric assessments of the concentrations of multiple essential nutrients in sweat [8]. Noura et al. presented a paper-based microfluidic electrochemical integrated device for measuring the glucose concentration in sweat in real time [9].

Most of the systems intended to analyze the content of sweat were tested while performing a physical activity, such as cycling. This method was used to validate the developed systems because it is an easy way to obtain significant volumes of this biofluid passively. During physical activity, sweat is generated to regulate the body’s internal temperature. The analysis of sweat at the time of an activity allows sports medicine doctors to assess an athlete’s performance and state of health.

It has been demonstrated that the monitoring of physiological parameters using wearable devices during a physical activity helps to prevent injuries and enhance the performance of athletes [10]. However, mainly the heart rate (HR) is used by sport professionals to determine the state of the athlete during the exercise [11]. Other parameters that give a lot of information, such as the blood lactate level and user’s dehydration , are rarely measured, and when they are monitored, a special test must be carried out.

In this paper, we present a novel wearable device capable of collecting sweat from the skin, canalizing it inside a microfluidic chamber, and analyzing its content using an array of sensors. The device was based on a sport chest strap that was modified to integrate the microfluidic system, the sensors, and the device’s electronics. This system is intended for estimating the user’s heart rate, dehydration, and blood lactate levels during physical activity. This paper focuses on the development of the system, sensors, and involved electronics and the initial results. Materials and methods for the obtention of blood lactate levels and dehydration state from the data collected are not developed here.

## 2. Materials and Methods

### 2.1. System Architecture

**Figure 1:**
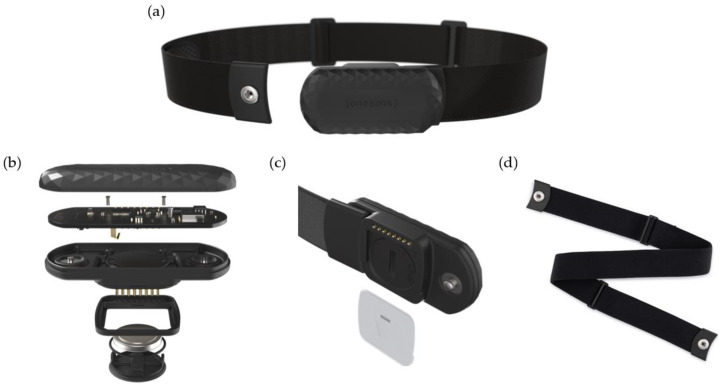
Structure of the wearable device. (**a**) Complete system, (**b**) central module (electronics of the system), (**c**) cartridge, and (**d**) chest strap.

The wearable device (Figure 1a) was conceived as a band that can be placed on the athlete’s chest. This system is formed by three different elements: a central module (Figure 1b), a cartridge (Figure 1c), and a chest strap (Figure 1d).

**Figure 2:**
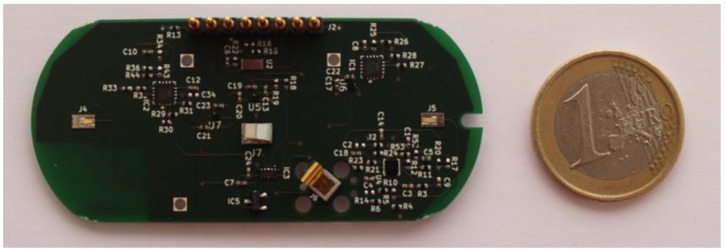
Photograph of the electronics of the device.

The central module contains the electronics of the system (Figure 2). These electronics capture the response of the device’s sensors by means of a custom analog front-end, process the raw data obtained from the sensors, and send the results to a mobile phone application via Bluetooth Low Energy (BLE). The entire system is controlled by a STM32L476JEY6TR microcontroller (STMicroelectronics; Geneva, Switzerland).

To power the system, a lithium coin cell battery with a nominal voltage of 3.0 V and a rated capacity of 210 mAh is used [12]. The voltage is regulated to 3.0 V, and this is the operational voltage of the system.

**Figure 3:**
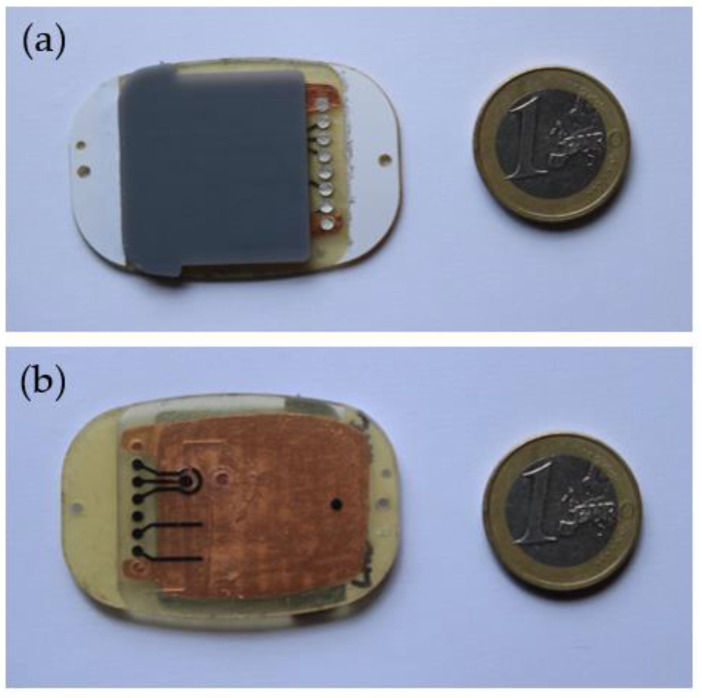
Image of the cartridge. (**a**) Front and (**b**) back of the disposable.

The cartridge (Figure 3) is the element that collects sweat from the skin and canalizes it into a microfluidic channel. There are several sensors inside this channel that analyze the content of this biofluid. This cartridge is a single-use component (disposable) that must be changed after each test. However, continuous measurements can be carried out for a given test, obtaining the evolution profile of every parameter during the exercise.

The chest strap provides a comfortable and robust attachment to the body and is utilized to perform HR measurements. This measurement is possible thanks to two rubber electrodes integrated inside the band.

**Figure 4:**
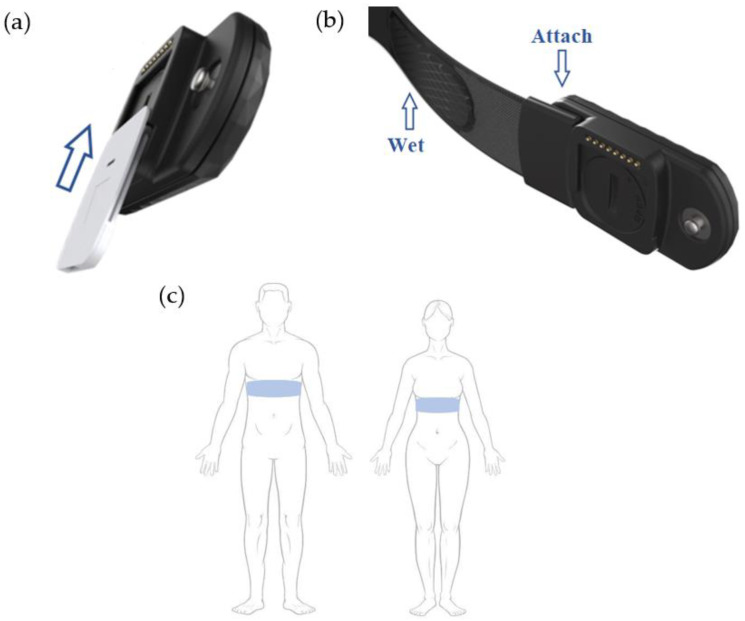
How to connect the different elements of the wearable device. (**a**) First, peel the protective layer and slide the cartridge into the central module (as is indicated by the arrow). This procedure ensures that the connections are isolated from the environment and the cartridge is robustly attached during the exercise. (**b**) Then, clip the strap to the central module and (**c**) put it on the chest. At the end, attach the cartridge to the skin. To remove the disposable, detach the adhesive from the skin and slide down the cartridge.

All the mentioned elements of the wearable device must be connected together in order to take measurements. The chest strap and the cartridge are attached to the central module (Figure 4).

The system has two operational modes: low-power and measurement states. Initially, the system remains in the low-power mode until the athlete wears the device. Upon detection by a skin detector system, the device switches to measurement mode. In this state, the wearable device records the heart rate dynamics and analyzes the sweat content. Once the activity is completed, it returns to the low-power state.

The device consumes 85 µA and 5 mA in low-power and measurement modes, respectively. This device is capable of disabling and enabling different modules of the system in order to reduce consumption. If the user uses the device for one hour per day, the expected battery life is 30 days.

### 2.2. Chest Strap and Cartridge

The sensing elements of the wearable device are the chest strap and the sensors integrated inside the cartridge. The chest strap is employed for HR measurement, while the sensors within the disposable are utilized to estimate the sweat content.

#### 2.2.1. Chest Strap

The chest strap has two rubber electrodes that are in contact with the athlete’s skin. These electrodes are used to record the athlete’s cardiac electrical activity by measuring the difference in potential between the electrodes. An electrocardiogram (ECG) is a plot of the electric activity recorded during this process. The signals of an ECG have multiple waves that reflect the heart state. The P wave represents the depolarization of the atria, the QRS complex represents the depolarization of the ventricles, and the T wave represents the repolarization of the ventricles [13,14].

Rubber electrodes are typically utilized in sports bands because they are reusable and washable. The ECG recorded using these electrodes lacks the necessary quality to be used in clinical settings, but the QRS complex can be identified easily in the signals obtained. This complex indicates that one beat has taken place, so it can be used to estimate HR. HR is the number of heartbeats per unit of time, usually per minute.

The system presented in this paper utilized the chest strap from the heart rate monitor TICKR product (Wahoo Fitness; Atlanta, GA, USA) [15].

#### 2.2.2. Cartridge

The cartridge is an adhesive patch attached directly to the user’s skin as well as to the central module of the device. This patch collects sweat from the skin and canalizes it inside a microfluidic channel. There are different sensors in this channel that are used to estimate the dehydration and the lactate levels of the athlete. The disposable design is not described here because it is outside the scope of this paper, and only the sensors are presented.

Dehydration is the condition that occurs when the body loses too much water. It can be caused by several reasons, such as sweating too much or not drinking enough water. If left untreated, this condition can lead to poor body function and, in extreme cases, death [16,17,18].

During physical activity, water is lost due to thermoregulatory sweating. The sweat glands of the body collectively secrete between 0.8 and 1.5 L of water in the course of a moderate-intensity workout [19,20]. This loss of fluids can lead to dehydration in the athlete, and it also produces an imbalance of body salts, since a lot of ions are lost through sweat [21].

Two different sensors are used to estimate the athlete’s dehydration. These sensors are a capacitive sensor [22,23] utilized to know the perspiration rate of the athlete, and a conductometric sensor used to estimate the sweat ionic strength.

**Figure 5:**
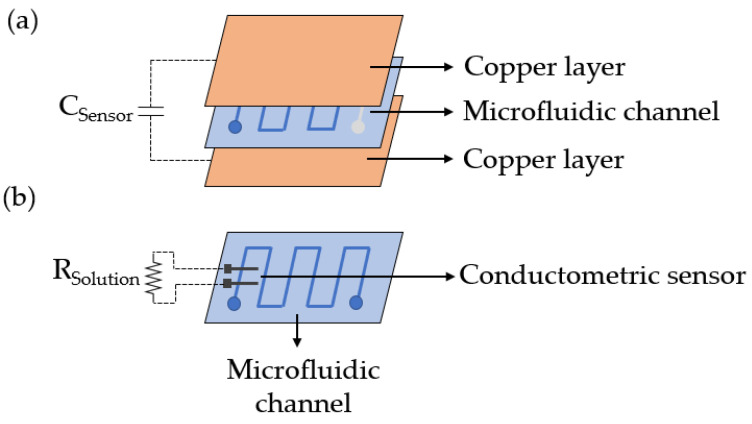
Dehydration sensors. (**a**) Scheme of the capacitive and (**b**) the conductometric sensors, respectively.

The capacitive sensor (Figure 5a) is formed of two layers of copper and a microfluidic channel that it is placed in the middle of the two metal sheets. At the beginning of each trial, the chamber is full of air, and once the test initiates, the channel starts to be filled with sweat. When the content inside the chamber changes, the dielectric constant of the sensor varies as well. This variation is translated to a modification of the system capacitance, so the volume changes within the channel can be detected by measuring this parameter. The volume monitored during a certain period of time can be related to the total volume of fluid lost by the athlete, and thus, the dehydration level can be estimated.

The ionic strength sensor (Figure 5b) is formed of two screen-printed electrodes integrated inside the microfluidic channel. These electrodes are in direct contact with the sweat that flows in the channel, and they are used to measure the impedance of this fluid. The main contribution of sweat impedance is the resistance of this biofluid, which depends on the number of ions present in the medium. Therefore, the concentration of ions can be estimated by measuring the conductivity of this biofluid. Specifically, the sodium concentration can be easily estimated, as it is the most abundant ion in sweat and dominates the conductivity response [24].

Lactate is a molecule produced through the breakdown of glucose under anaerobic conditions. Elevated blood lactate levels indicate an oxygen deficiency in some tissues of the body. During exercise, lactate is generated because there is insufficient oxygen reaching the muscles. The increase in lactate implies a higher concentration of protons within the cells, so if the lactate production rate is high, it may exceed the buffering capacity of protons and cause a decrease in pH, generating lactic acid that affects the performance of muscles [25,26,27].

The blood lactate concentration varies from 0.6 to 2.0 mM, but it can increase up to 10 mM during intense physical effort [28]. Many factors affect sweat lactate concentration, but it is estimated that it varies between 1 mM and 100 mM during physical activity [29,30]. Currently, lactate tests need a blood sample, generally taken from the earlobe through a needle, that causes a great disadvantage in real-time monitoring. Using this method can be difficult for taking a series of measurements during training and therefore obtaining follow-up results [31].

To know the relationship between the lactate levels in blood and sweat, a bioequivalence study was carried out by the authors of this work. The study is described in [32], which concludes that there is no direct equivalence between blood lactate and sweat lactate without considering other parameters. To estimate the equivalence, a custom algorithm that takes HR, perspiration level, and sweat lactate concentration as inputs is required.

There are different methods to estimate the concentration of a molecule, such as ultraviolet spectrophotometry or refractive index detection. However, these tests are time-consuming and very complex. Electrochemical biosensors have several advantages over traditional methods, like speed, high specificity, low cost, and possible miniaturization/integration into circuits to perform the measurement [33].

The lactate sensor used in this device is an enzyme-based amperometric sensor that combines the specificity and selectivity of enzymes with the high sensitivity of electrochemical sensing. This sensor consists of a working electrode made of a mixture of carbon and Prussian blue mediator, a silver reference electrode, and a carbon counter-electrode. These electrodes are screen-printed on the surface of a PET foil. The working electrode is functionalized with lactate oxidase, which is immobilized in a biopolymeric membrane whose function is to retain the enzyme on the electrode’s surface.

### 2.3. Electronics of the System

The disposable and the chest strap are attached to the central module of the device which contains the system electronics. The electronics capture the response of every sensor using custom analog instrumentations and the ADCs of the microcontroller.

#### 2.3.1. ECG Recording and Heart Rate Estimation

To record the user’s ECG, a commercial integrated AD8233 circuit (Analog Devices; Wilmington, MA, USA) is utilized. This chip is an analog front-end for the signal conditioning of cardiac biopotentials. It is composed of an instrumentation amplifier, an operational amplifier, and a right leg drive amplifier. The component also incorporates leads-on or -off detection circuitry.

Two different filters are implemented to eliminate the noise present on the ECG signal. The first filter is a first-order high-pass filter with a cutoff frequency of 7 Hz, and it is used to delete motion artifacts and the electrode half-cell potential. The second filter is a second-order low-pass filter with a cutoff frequency of 25 Hz, and it is utilized to remove other interference signals.

The Pan–Tompkins algorithm is used to calculate the HR from the ECG recorded by the analog front-end [34]. To estimate the HR, windows of 5 s of the ECG recording are utilized. The current consumption of this module is 150 µA at 3.0 V, when it is activated.

#### 2.3.2. Sweat Rate and Conductivity Instrumentations

As mentioned before, a capacitive sensor is used to estimate the user’s perspiration level, and a conductivity sensor is used to know the ionic strength of the sweat.

**Figure 6:**
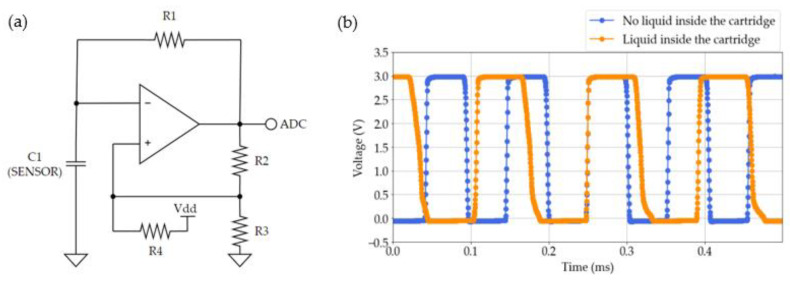
Modified Schmitt Trigger Oscillator. (**a**) Schematic of the circuit and (**b**) response of this instrumentation. The output signal is highlighted in blue when the disposable is empty, and in orange is the response of the instrumentation when the cartridge is full of fluid. The first signal has a period of 103 µs and the second one of 141 µs.

On one hand, a modified Schmitt Trigger oscillator is utilized to measure the response of the capacitive sensor (Figure 6a). This circuit is based on the operational amplifier TSX564IQ4T (STMicroelectronics; Geneva, Switzerland). The oscillator generates a square signal whose frequency depends on the capacitance of the system (Figure 6b). This capacitance is the sensor, so the changes in the period of the signal are directly related to the variation in the sensor impedance. As was mentioned before, the perspiration sensor modifies its capacitance as sweat enters inside the microfluidic channel, so the changes in volume inside this channel can be easily detected.

**Figure 7:**
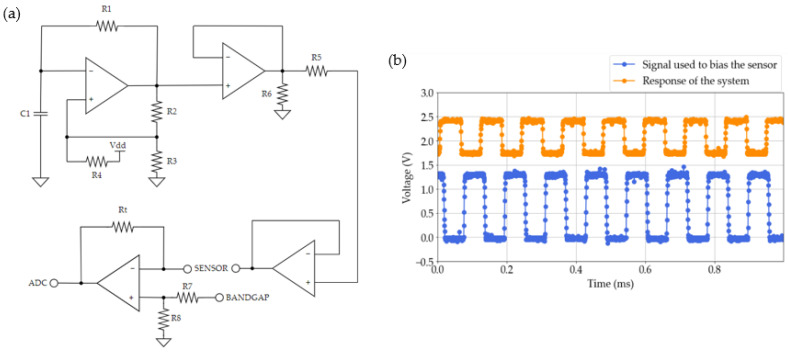
Conductivity instrumentation. (**a**) Schematic of the instrumentation and (**b**) response of this circuit. The signal used to bias the sensor is shown in blue, while the system’s response to a conductive fluid inside the disposable is indicated in orange. The amplitude of the signal provided by the instrumentation changes depending on the medium conductivity.

On the other hand, a circuit that combines the modified Schmitt Trigger oscillator, an amplification stage, and a transimpedance amplifier is used to quantify the sweat ionic strength (Figure 7a). This circuit uses the same operational amplifier as in the previous case. It generates a square signal of 8 kHz using the mentioned oscillator. Then, the amplitude of this signal is reduced by the amplification stage in order to not saturate the conductivity sensor. Finally, this signal is applied to the sensor, and the response is captured by a transimpedance amplifier (Figure 7b). The bandgap reference REF2920AIDBZR (Texas Instruments; Dallas, TX, USA) is used to provide a bias in order to adjust the signal to the ADC levels of the microcontroller. From the amplitude of the signal acquired, the conductivity of the biofluid is estimated. To obtain this value, the cell constant of this sensor is utilized. This constant depends on the geometry of the sensor, and it defines the resolution of the measurement [35]. Once the conductivity is calculated, the number of ions present in this biofluid can be estimated.

The current consumption of the first module is 650 µA at 3.0 V and the second one is 800 µA at the same operating voltage when they are activated.

#### 2.3.3. Potentiostat

A potentiostat is an electronic instrument used to carry out several electrochemical techniques, such as chronoamperometry and cyclic voltammetry. It is a circuit that biases the electrochemical cell under study and captures its response [36].

**Figure 8:**
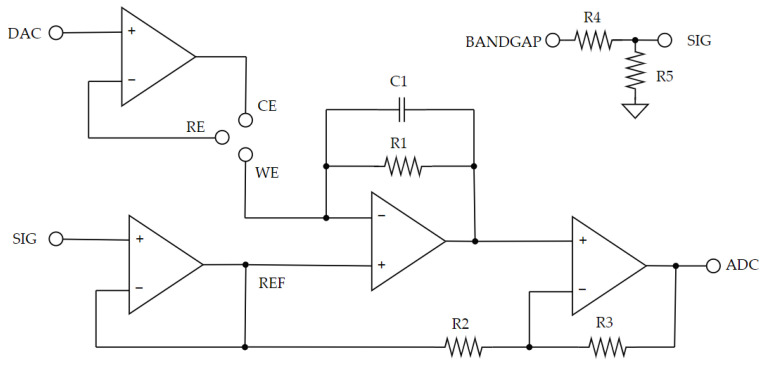
Potentiostat used to bias the lactate sensor and capture its response.

A custom potentiostat based on the operational amplifier ADA4505-4 (Analog Devices; Wilmington, MA, USA) is utilized (Figure 8). This potentiostat is designed to work along three electrode amperometric sensors. Its architecture is based on a transimpedance amplifier with a direct voltage bias application and a final non-inverting amplifier, as low current levels are expected from the lactate sensor. Moreover, a capacitor is introduced as a first-order low-band-pass filter. This potentiostat can provide a whole voltage supply compliance voltage as well as read both oxidation (negative current) and reduction currents (positive current) from the amperometric sensor. It is complemented by the same bandgap used for the conductometric instrumentation and the microcontroller’s DAC, which provides the necessary voltage bias to the sensor. The complete characterization of this potentiostat can be found in article [37].

## 3. Results and Discussion

The system was validated by means of several in vitro and in vivo tests. In vitro trials ensured good functioning of each measurement and in vivo test demonstrated their functionality in a real environment.

### 3.1. In Vitro Tests

Each sensor and instrumentation was tested individually and validated by comparing the data obtained using the gold-standard method of each type of measurement.

To characterize the HR parameter, a specific protocol was created. This protocol consisted of an equivalence study between the wearable device and the sports band Suunto Smart HR Belt (Suunto; Vantaa, Finland) [38]. It has been demonstrated that this kind of band has an accuracy similar to an ECG Holter monitor [39]. The protocol of this study is as follows:

- Moisten the rubber electrodes of the wearable device and the reference band, before placing them on the chest of a volunteer.
- Collect the data from each device using a mobile phone application, while the volunteer is sitting, for five minutes.
- Collect the data from each device using a mobile phone application, while the volunteer is pedaling on a stationary bicycle at a constant load, for five minutes.
- Collect the data from each device using a mobile phone application, while the volunteer is sitting, again, for five minutes.

Using this protocol, eight tests were performed, and the results of these trials were analyzed. The volunteers of this study included five males and three females with ages ranging from 21 to 39 (mean of 28.6 years and standard deviation of 5.9 years). Both the correlation coefficient and the mean standard deviation were utilized to determine the accuracy of this measurement (Table 1). The correlation coefficient is close to one, so the HR obtained by the wearable device shows no significant difference when compared with the data provided by the Suunto Smart HR Belt. Additionally, the mean standard deviation is less than three beats per minute (bpm), indicating low measurement dispersion.

To validate the ionic strength measurement, a direct comparison was carried out bet-ween the wearable device and the reference equipment, LAQUAtwin EC-11 (Horiba; Kioto, Japan) [40]. The analyte utilized to perform this analysis was artificial sweat. This artificial sweat was prepared using deionized water with 0.5% sodium chloride, 0.1% potassium chloride, 0.1% lactic acid, and 0.1% urea. From this solution, ionic strength dilutions of 10, 30, 50, 70, 100, and 130 mM were made.

**Figure 9:**
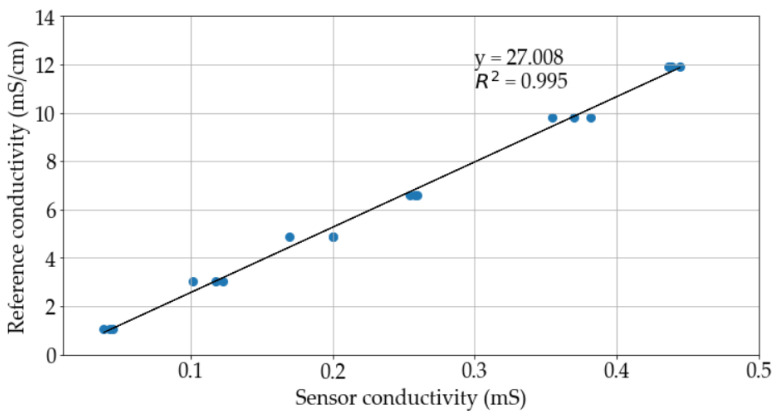
Fit between the conductivity calculated by the wearable device and the reference equipment. The dots correspond to each measurement carried out by the wearable device.

Multiple measurements were taken using three different conductometric sensors. A linear regression was employed to compare the performance of the device with the reference equipment (Figure 9). The coefficient of determination of 0.995 shows a good fit between the data, so the system can be considered accurate.

To validate the perspiration level measurement, no reference equipment was used because there does not exist standard instrumentation to estimate the sweat rate. Instead, a KDS Legato 101 syringe pump (KD Scientific; Holliston, MA, USA) was utilized [41] to simulate the perspiration of the user. This element continuously pumped artificial sweat inside the disposable at a fixed rate of 2 µL/min.

**Figure 10:**
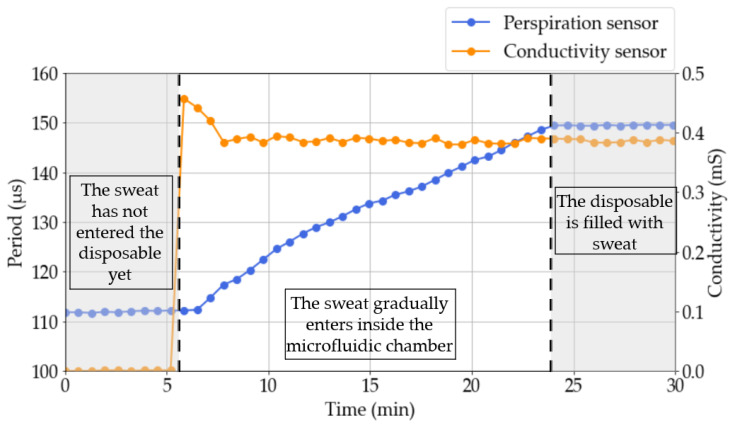
Conductivity and sweat rate obtained during an in vitro test.

A trial of 30 min was performed using this syringe pump and a cartridge. During this test, both sweat rate value and conductivity were recorded (Figure 10). The response of the capacitive and the conductometric sensors remained constant at the beginning of the test since the fluid had not yet entered inside the microfluidic channel. When the liquid entered the chamber, the conductometric sensor gave an unstable output that was stabilized at a fixed value after 2 min. The sweat rate sensor was activated immediately, and it gave a response that is linearly time dependent. Finally, when the microfluidic channel was full of liquid, the sensor response stayed constant because the system capacitance could no longer change.

**Figure 11:**
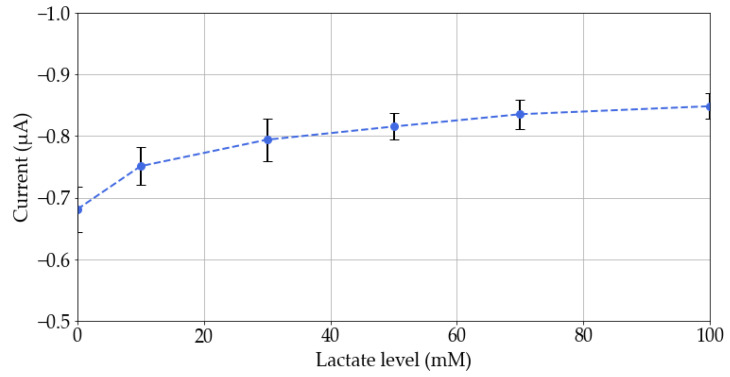
Response of each sensor and potentiostat to different concentrations of lactate. The error bars correspond to the standard deviation between measurements of multiple sensors.

Eventually, the lactate measurement was characterized using artificial sweat. This solution was modified to obtain 0, 10, 30, 50, 70, and 100 mM lactate concentrations. Different measurements with three different lactate sensors were made using the custom potentiostat. Chronoamperometries of 60 s using a working potential of −0.3 V were performed. Only the last value was taken because it is the moment when the sensor stabilizes its response (Figure 11). No comparison with reference instrumentation was carried out since the characterization of this potentiostat can be found in [37].

A trend in the system’s response is observed: the current produced by the sensor increases as the lactate concentration rises. This response allows to monitor the level of this molecule and predict the sweat lactate concentration.

### 3.2. In Vivo Tests

Once both the sensors and the electronics were validated in vitro, the system was ready to be tested in vivo. This test consisted of the utilization of the wearable system to analyze the sweat content of volunteers during physical activity.

All the participants of the study were informed and gave their consent to use the data extracted from the experiments. The volunteers included two males and one female. Before starting the test, the participants were asked to provide the researchers with a series of personal data (Table 2).

The in vivo testing protocol is as follows: 

Clean with water the skin area where the disposable is placed, and then, dry the area to ensure that the disposable’s adherence to the skin is good.

**Figure 12:**
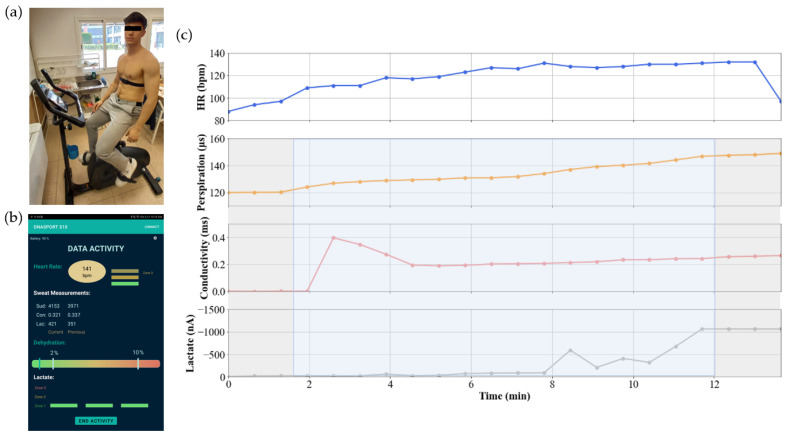
Real-time perspiration analysis during stationary cycling. (**a**) Photograph of the experimental set-up and (**b**) the application used to capture the data produced by the wearable device. (**c**) Results obtained via one in vivo trial. In gray are highlighted the moments when the cartridge is empty and full. The time in which the cartridge is filled is marked in blue.

- Attach the heart rate monitor strap to the central module and wet both rubber electrodes.
- Remove the adhesive liner of the disposable and adhere the central module carrying the cartridge to the skin.
- Collect the data from the wearable device while the volunteer is pedaling on a stationary bicycle at a constant load (Figure 12a). All the data are gathered using a mobile application, and these data are downloaded as a CSV file (Figure 12b).

The results of the in vivo trials were the expected ones for every partaker (Figure 12c). At the beginning of the test, the HR increased until it reached its maximum value and remained stable. When the HR value increased, the perspiration of the athlete began. A few minutes passed until the cartridge started to collect sweat from the skin, as is observed in the response of the conductometric sensor. This sensor gave an initial peak that can be associated with the trapped minerals (iron, zinc, magnesium, etc.) in the sweat duct [20]. The progressive increase when the activity continued could be explained by the dependence on sweat rate (reabsorption channels in the sweat duct) [5]. More importantly, these results mean that the microfluidic system associated with the sensor was making the sweat continuously circulate, and was able to determine the chronological evolution of the different biomarkers. Meanwhile, the capacitive sensor gave an increasing response over time until the microfluidic channel was full of liquid. Eventually, the lactate sensor started its measurement by giving an increasing output until it stabilized its response at a fixed value after a few minutes elapsed.

The behavior of all these sensors was almost the same as in the in vitro trials. Only small changes were observed in the response time and the absolute value of the sensors. The first difference was expected since the perspiration rate of the in vivo test was not constant over time and every volunteer had different perspiration profiles. On the other hand, the second difference can be associated with the sweat composition. Artificial and real sweat are not the same, so different responses were expected. Sweat contains a large quantity of ions that modify the properties of the medium. Enzymatic electrochemical sensors are affected a lot by environmental conditions. In this case, the saltiness of the medium interacted with the activity of the lactate sensor enzyme, producing higher current levels than expected.

The outcomes of these trials can be considered a great success since real-time sweat analysis was performed. Despite this fact, more work needs to be carried out in order to fully understand the obtained results. To achieve this, more in vivo tests must be performed and the physiology of perspiration must be studied.

The microfluidic system defines the capabilities of the cartridge in terms of sensor response time and durability. This could be adapted by modifying the collection area, which determines the total fluid volume, and the configuration of the microfluidic channels to maximize volume capability.

Eventually, improvements in the consumption of the system can be made, and additional measures such as heart rate variability (HRV) can be added. HRV is the variance in time between heart beats and it is regularly used in the athletic world to identify periods of optimal training and to monitor recovery status and any potential overtraining [42].

## 4. Conclusions

In this paper, we present a groundbreaking advancement in the field of sports monitoring: a wearable device designed for continuous athlete monitoring in real time through sweat analysis. This innovative system utilizes a conventional sports band that incorporates a single-use cartridge capable of collecting, processing, and analyzing sweat from the user’s skin, all thanks to an array of sensors integrated into this disposable. What makes our approach exceptional is its ability to provide an instant assessment of the athlete’s health without disrupting their routine. This device is not only non-intrusive, but is seamlessly integrated into the user’s daily life, making adoption effortless.

Every component of the device has undergone rigorous testing, both in vitro and in vivo. Promising results that open the door to an exciting future for health monitoring in sports have been achieved. However, the true potential of this system lies in its ability to translate the collected data into valuable insights for athletes. This means that there is still much to be done in order to interpret these data and provide athletes with a deep understanding of their performance and health. To interpret these data, dozens of trials with multiple athletes must be performed to create a large database and analyze all the collected data.

Apart from enhancing the daily lives of athletes, this technology has the potential to play a crucial role in the medical field. Non-invasive and continuous measurements of sweat composition can provide healthcare providers with information about biochemical parameters, such as glucose level, that could allow them to improve the attention given to patients and their treatment. A direct application of this system is the assessment of cystic fibrosis since the conductivity of sweat rises a lot when a user has this disease. Despite this fact, to translate this device from the sports to the medical field, a lot of work is required in the field of sensing and microfluidics in order to enable it to process low volumes of sweat.

## Figures and Tables

**Table 1:** Statistical analysis of the comparison between the HR calculated by the wearable device and the reference instrument.

Participants	Correlation Coefficient	Mean Standard Deviation
Males	0.81	2.9 bpm
Females	0.83	2.8 bpm

**Table 2:** Characteristics of the volunteers of the in vivo tests.

Parameters	Volunteer 1	Volunteer 2	Volunteer 3
Sex	Male	Female	Male
Age (years)	30	21	39
Height (cm)	175	164	176
Weight (kg)	80.4	52.0	75.0
BMI (kg/m^3^)	26.3	19.3	24.2

## Data Availability

Data is contained within the article.
